# Assessment of Efficacy and Quality of Two Albendazole Brands Commonly Used against Soil-Transmitted Helminth Infections in School Children in Jimma Town, Ethiopia

**DOI:** 10.1371/journal.pntd.0004057

**Published:** 2015-09-25

**Authors:** Sileshi Belew, Mestawet Getachew, Sultan Suleman, Tesfaye Mohammed, Habetewold Deti, Matthias D'Hondt, Evelien Wynendaele, Zeleke Mekonnen, Jozef Vercruysse, Luc Duchateau, Bart De Spiegeleer, Bruno Levecke

**Affiliations:** 1 School of Pharmacy, Jimma University, Jimma, Ethiopia; 2 Drug Quality and Registration Group, Department of Pharmaceutical Analysis, Faculty of Pharmaceutical Sciences, Ghent University, Gent, Belgium; 3 Jimma University Specialized Hospital, Jimma University, Jimma, Ethiopia; 4 Department of Medical Laboratory Sciences and Pathology, Jimma University, Jimma, Ethiopia; 5 Department of Virology, Parasitology and Immunology, Faculty of Veterinary Medicine, Ghent University, Merelbeke, Belgium; 6 Department of Comparative Physiology and Biometrics, Faculty of Veterinary Medicine, Ghent University, Merelbeke, Belgium; Fundación Mundo Sano, ARGENTINA

## Abstract

**Background:**

There is a worldwide upscale in mass drug administration (MDA) programs to control the morbidity caused by soil-transmitted helminths (STHs): *Ascaris lumbricoides*, *Trichuris trichiura* and hookworm. Although anthelminthic drugs which are used for MDA are supplied by two pharmaceutical companies through donation, there is a wide range of brands available on local markets for which the efficacy against STHs and quality remain poorly explored. In the present study, we evaluated the drug efficacy and quality of two albendazole brands (Bendex and Ovis) available on the local market in Ethiopia.

**Methodology/Principal Findings:**

A randomized clinical trial was conducted according to the World Health Organization (WHO) guidelines to assess drug efficacy, by means of egg reduction rate (ERR), of Bendex and Ovis against STH infections in school children in Jimma, Ethiopia. In addition, the chemical and physicochemical quality of the drugs was assessed according to the United States and European Pharmacopoeia, encompassing mass uniformity of the tablets, amount of active compound and dissolution profile. Both drugs were highly efficacious against *A*. *lumbricoides* (>97%), but showed poor efficacy against *T*. *trichiura* (~20%). For hookworms, Ovis was significantly (p < 0.05) more efficacious compared to Bendex (98.1% *vs*. 88.7%). Assessment of the physicochemical quality of the drugs revealed a significant difference in dissolution profile, with Bendex having a slower dissolution than Ovis.

**Conclusion/Significance:**

The study revealed that differences in efficacy between the two brands of albendazole (ABZ) tablets against hookworm are linked to the differences in the *in-vitro* drug release profile. Differences in uptake and metabolism of this benzimidazole drug among different helminth species may explain that this efficacy difference was only observed in hookworms and not in the two other species. The results of the present study underscore the importance of assessing the chemical and physicochemical quality of drugs before conducting efficacy assessment in any clinical trials to ensure appropriate therapeutic efficacy and to exclude poor drug quality as a factor of reduced drug efficacy other than anthelminthic resistance. Overall, this paper demonstrates that “all medicines are not created equal”.

## Author Summary

Soil-transmitted helminths (STHs) infect millions of children worldwide. To fight STH, large-scale de-worming programs are implemented in which anthelmintic drugs (either albendazole (ABZ) or mebendazole (MEB)) are administered. However, there is a wide range of other brands, which are even more accessible, but for which the efficacy and quality remain poorly explored. We evaluated efficacy against STHs and quality of two ABZ brands commonly available on the local markets in Ethiopia (Bendex and Ovis). Both brands showed high efficacy against roundworm infections and poor efficacy against whipworms. However, for hookworm infections, Bendex was significantly less efficacious than Ovis. In terms of drug quality, a significant difference was observed in the dissolution profile, with Bendex having a significantly slower dissolution rate than Ovis. Since dissolution behavior is critical for a drug to be appropriately absorbed into the helminth (through host-blood and/or parasite-cuticle) and produce therapeutic efficacy, the poor dissolution of Bendex compared to Ovis can explain the observed difference in efficacy against hookworms. Our results emphasize the importance of periodically assessing of drug quality to ensure appropriate therapeutic efficacy and to exclude poor drug quality as a potential factor of reduced drug efficacy other than drug resistance.

## Introduction

Currently, there is a worldwide upscale in the implementation of programs to control and to eliminate a selection of 10 neglected tropical diseases [1, 2]. Among these, soil-transmitted helminthiasis causes the highest burden on public health. It is estimated that more than 1.4 billion people were infected with at least one of the four STH species: the roundworm *Ascaris lumbricoides*, the whipworm *Trichuris trichiura* and the two hookworm species *Necator americanus* and *Ancylostoma duodenale*, resulting in a global burden of approximately 5.2 million disability-adjusted life years (DALYs) (20% of the total number of DALYs attributable to neglected tropical diseases) [3, 4]. To control the morbidity caused by STH, mass drug administration (MDA) of a single oral dose of a benzimidazole anthelminthic drug (ABZ or MEB) is recommended in communities where the prevalence of any STH exceeds 20% [5].

To date, major pharmaceutical companies such as GlaxoSmithKline (ABZ, Zentel) and Johnson and Johnson (MEB, Vermox) are donating these medicines to WHO, which subsequently distribute these medicines to its recipient countries. In Ethiopia, the donated medicines are made available for the patients through government hospitals and health centers. The therapeutic efficacy of these products at the WHO-recommended dosages (*i*.*e*. single dose of 400 mg ABZ or 500 mg MEB) has recently been evaluated in two consecutive multinational trials [6, 7]. These trials showed that the therapeutic efficacy measured in terms of egg reduction rate (ERR), varied both between drugs and STH species: both drugs showing high efficacy against *A*. *lumbricoides* (> 98%) and poor efficacy against *T*. *trichiura* (~64%), and ABZ being more efficacious against hookworms compared to mebendazole (96% *vs*. 80%). In addition to these two donated brands, there is a wide range of other brands available on local markets of STH endemic countries, which are often more accessible to the local people, but for which the efficacy or quality remain poorly explored [8]. The latter is particularly important in countries where resources are limited to monitor the quality of drugs, and hence in which prevalence of substandard, falsified or illegal drugs is substantial [9–14]. Although the quality of medicines has a direct influence on therapeutic efficacy, this remains poorly studied for benzimidazole anthelminthic drugs against STH infections. Therefore, we assessed both the efficacy and quality of two brands of ABZ commonly administered for the treatment of individual STHs in Ethiopia, namely Bendex and Ovis.

## Methods

### Ethical statement

The study protocol was approved by the Ethical Committees of Jimma University (Ethiopia) (reference no RPGC/282/2014) and of the Faculty of Medicine, Ghent University (Belgium) (ref. no 2013/1114; B670201319330). The study is registered under clinicaltrial.gov identifier number NCT02420574 (https://clinicaltrials.gov/ct2/show/NCT02420574?term=NCT02420574&rank=1). The school authorities, teachers, parents, and the children were informed about the purpose and procedures of the study. The written consent form was prepared in two commonly used local languages (Afaan Oromo and Amharic) and handed over to the children’s parents/guardians after explaining the aim, confidentiality and entire procedure of the clinical trial. Only those children (i) who were willing to participate and (ii) whose parents or guardians signed the written informed consent form were included in the study. Moreover, an additional separate written informed consent form for children older than 12 years was prepared, read and handed over to them and their additional written informed consent obtained (S1 Checklist).

### Origin of the drugs

Samples of the two ABZ brands (Bendex, India, CIPLA Ltd, batch no: x21253 and Ovis, Korea, DaeHWa Pharmaceuticals, batch no: 2020) with a label claim of 400 mg/tablet and expiry date of November 2015 were purchased from private community pharmacy in Jimma town, Ethiopia.

### Assessment of therapeutic efficacy against STH infections in school children

#### Study site and study population

The study was conducted in Jimma town, Ethiopia, which is located approximately 350 km southwest of the capital Addis Ababa. This study focused on school children (aged 5 to 18 years) from 2 out of the 24 primary schools that are found in Jimma Town. These schools were selected based on previous STH prevalence data [15, 16] and their involvement in previous drug efficacy trials [6, 7, 17]. At the time of this trial, these schools were not included in any MDA program.

#### Study design

**Fig 1:**
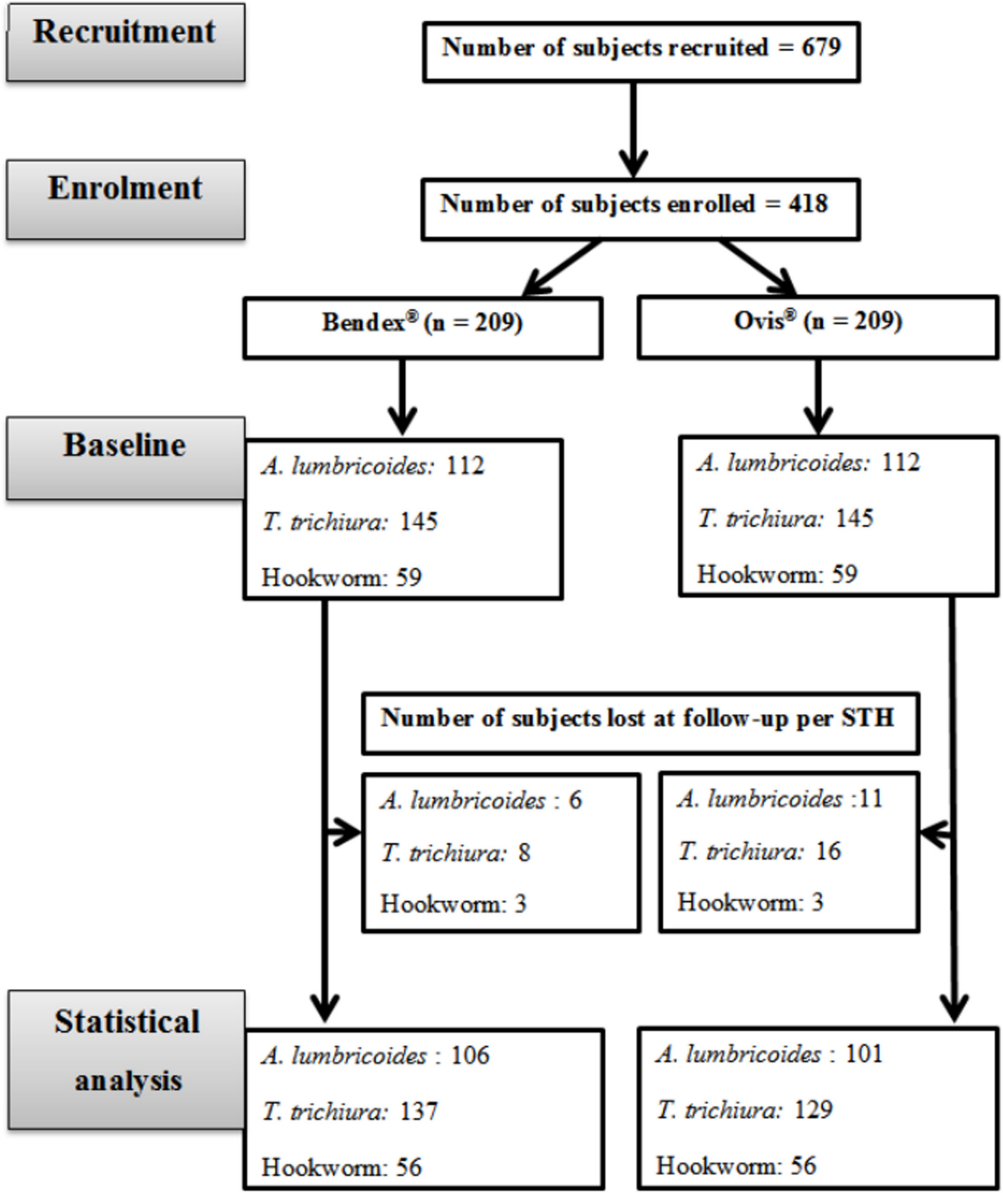
The number of subjects recruited, enrolled, and lost at follow-up in a two-armed efficacy trial.

The initial objective of this study was to assess the efficacy of ALB medicines against STH infections in school children. To this end, we applied the recently published WHO guidelines on the efficacy assessment of anthelminthic drugs against Schistosomiasis and STH to evaluate the efficacy of both brands [5]. In this guideline, at least 50 infected subjects per STH species are required. We also evaluated the quality of both medicines. Hence, this survey was originally not designed to assess a difference in ERR between brands, but merely to verify whether drugs were satisfactory, doubtful or unsatisfactory. Hence, no a prior power calculation was performed, but the results were repeated together with the 95% CI and p-value (at significant level of 5%). In short, parents/guardians were informed about the aims and the procedures of the entire clinical trial. Based on this, school children were recruited on a voluntary basis and asked to provide a stool sample during a pre-intervention survey. Among these, infected children who fulfill the WHO criteria for efficacy trial were enrolled for intervention. For the initial sampling, the aim was to enroll at least 100 infected children (50 for each ABZ brand) for *A*. *lumbricoides*, *T*. *trichiura* and hookworm, separately. Subjects with multiple infections were subsequently randomized across the two ABZ brands, stratifying for mono, double and triple STH infections. The drugs were administered under the direct supervision of a clinical nurse. Fourteen days after drug administration, stool samples were again collected from the subjects. All stool samples were processed by the McMaster egg counting method [18]. A tutorial on how to perform the McMaster egg counting method can be found at http://www.youtube.com/watch?v=UZ8tzswA3tc. Subjects who were unable to provide a stool sample at follow-up, or who were experiencing a severe intercurrent medical condition (any medical conditions other than parasitic infection) or had diarrhea at the time of the first sampling, were excluded from the study. The flowchart summarizing the number of subjects recruited, enrolled, lost at follow-up and included in the statistical analysis is presented in Fig 1, bearing in mind that individual children may have double infections.

### Assessment of drug quality

The quality of the drugs was evaluated by investigating three efficacy-critical quality attributes: (i) the mass uniformity, (ii) the amount of the active compound, and (iii) the dissolution of the tablets.

#### Drugs/reagents/chemicals/solvents

ABZ USP reference standard (Greenfield Pharmaceuticals, batch number: 20070504), Sulfuric acid AR (reagent chemicals service Ltd, United Kingdom), ammonium phosphate monobasic (extra pure, Cisco Research laboratory Pvt. Ltd., India), hydrochloric acid (36% w/v, Fisher Chemicals, Germany), sodium hydroxide (BDH Laboratory supplies, England), HPLC grade methanol and acetonitrile (CARLO ERBA Reagents, England) and ultrapure HPLC water (18.2 MΩ cm resistivity) were used.

#### Mass uniformity

Twenty tablets of each brand were randomly selected and individually weighed with a calibrated balance (Metter Toledo, AL204-1C, Switzerland) with experimentally determined operating range of 94.63 mg to 200 g and accuracy of 0.006% [19]. This mass uniformity between tablets of each brand was evaluated against the European pharmacopoeia specification [20].

#### Amount of active compound

The assay of the active pharmaceutical ingredient (API) was performed by a validated reversed-phase method according to USP [21], using an Agilent 1260 HPLC system equipped with an Agilent ZORBAX SB-18 column (150 x 4.6 mm, 5 μm) and UV-VIS Diode Array Detector. The detection wavelength, column temperature, flow rate of the mobile phase and injection volume were set at 254 nm, 25°C, 1 ml/min and 20 μl, respectively. The isocratic mobile phase used was composed of methanol and 0.05 M phosphate buffer (pH 5.5) (60:40% v/v). The analytical method was validated according to International Conference on Harmonisation (ICH) Q2(R1) recommendation [22]. The linearity of the method was evaluated around the target label claim concentration (analytical aliquot concentration of 200 μg/ml) ranging from 160 to 240 μg/ml ABZ. The regression line was assessed by determining the 95% confidence interval (95% CI) of slope and intercept parameters as well as by evaluating the residual plot. Repeatability precision was evaluated by injecting six replicates of 100% test concentration (200 μg/mL) and percent relative standard deviation (%RSD) of the measurements was calculated. The accuracy and range of the method was determined by spike experiments at five different concentrations corresponding to 80, 90, 100, 110 and 120% of the nominal analytical concentration (200 μg/ml).

System suitability was evaluated by (1) the symmetry factor (A_s_) of the ABZ reference standard, calculated using the European Pharmacopoeia (Ph. Eur.) equation *A*_*s*_ = *w*_0.05_ / 2*d*, where, *w*_0.05_ is the width of the peak at one twentieth of the peak height and d is the distance between the perpendicular dropped from the peak maximum and the leading edge of the peak at one twentienth of the peak height; and (2) injection repeatability by injecting (6 times) the ABZ reference standard solution. The Ph.Eur. system suitability test (SST) specifications were considered, *i*.*e*. A_s_ maximally 1.5 and percentage relative standard deviation (%RSD) maximally 0.85 [23].

#### Dissolution profile

The dissolution of both brands was evaluated using a dissolution apparatus II (paddle) (RC-6D Tian Jin optical instruments, China) following United States Pharmacopoeia recommendation [21]. The dissolution medium (900 mL 0.1 N HCl) was transferred to vessels of dissolution apparatus and the temperature and the spindle rotation speed were set to 37 ± 0.5°C and 50 rpm, respectively. Three tablets from each brand were randomly assigned to the six dissolution vessels and 10.0 ml samples were withdrawn at predetermined time points (0, 15, 30, 35, 40, 45 min) and replenished with an equal volume of fresh dissolution medium at the same temperature. The samples were immediately filtered using Whatman No. 1 filter paper and suitably diluted in 250 mL volumetric flask with 0.1 N sodium hydroxide. The absorbance of each sample was determined at 308 nm and 350 nm using a UV/Visible spectrophotometer (Aquarius, Cambridge, England) and the difference taken as absorbance value. A 0.1 N sodium hydroxide was used as the blank solution. The quantity in milligram (mg) of ABZ dissolved was calculated by the formula 22.5*C*(A_u_/A_s_), where C is the concentration of ABZ reference standard (RS) in the standard solution (μg per mL) and A_u_ and A_s_ are the absorbance differences, obtained at 308 nm and 350 nm, of the solution under test (A_u_) and the standard solution (A_s_). This method was also validated according to ICH Q2(R1) recommendations, for linearity, precision and accuracy [22]. In order to clarify one of the possible reasons to explain the different dissolution behavior, IR spectra of both tablets were recorded on a qualified FTIR spectrometer (Thermo Scientific, USA), using the KBr-disk methodology as described in the Ph. Eur. [24].

### Statistical analysis

#### Assessment of drug efficacy

The efficacy of each ABZ brand was reported for each of three STH species by means of ERR, using the formula below:

$$\text{ERR}\;(\%)=100\%\left(\frac{\text{arithmetic mean}\;(\text{FEC at baseline})\;\text{- arithmetic mean}\;(\text{FEC at follow up})}{\text{arithmetic mean}\;(\text{FEC at baseline})}\right)$$

Based on the obtained ERR results, the efficacy of the brands were classified as ‘satisfactory’, ‘doubtful’ and ‘reduced’. The criteria applied for this classification were suggested by WHO [5] and are summarized in Table 1. The 95% confidence intervals (95% CI) for age, sex ratio and FEC at baseline, and ERR were determined by bootstrap analysis (10,000 iterations). Permutation tests were performed to verify differences between both groups [25]. These permutation tests consisted of 2 consecutive steps. First, the permutation distribution under the null hypothesis that in average there was no difference in age, sex ratio, and FEC at baseline, and ERR between two treatment arms was generated. To this end, all individuals of both arms were randomly re-assigned to one of the treatment arms (the number of individuals in each treatment arm remained unchanged). The new mean age, sex ratio, FEC and ERR for each of the two treatment arms was determined and the absolute value of the difference in age, sex ratio, FEC and ERR between treatment arms was calculated. Next, this procedure was repeated 10,000 times. The distribution of these 10,000 permutated age, sex ratio, FEC and ERR differences represented the permutation distribution when the null hypothesis is true. Second, the probability of finding a value as extreme as the absolute value of the observed difference in age, sex ratio, FEC and ERR between the two treatment arms in this permutation distribution was determined. The level of significance was set at p < 0.05.

**Table 1:** The criteria by WHO recommended to classify the efficacy of albendazole against soil-transmitted helminths.

Criteria	*A*. *lumbricoides*	*T*. *trichiura*	Hookworm
**Satisfactory**	ERR ≥ 95%	ERR ≥ 50%	ERR ≥ 90%
**Doubtful**	95% > ERR ≥ 85%	50% > ERR ≥ 40%	90% > ERR ≥ 80%
**Reduced**	ERR < 85%	ERR < 40%	ERR < 80%

### Assessment of drug quality

#### Mass uniformity

The uniformity of mass between tablets of each brand was evaluated against Pharmacopoeial specification limit.

#### Amount of active compound

The appropriateness of the model was assessed by determining the 95% confidence interval (95% CI) of slope and intercept parameters of regression line as well as by evaluating the residual plot. The amount of active compound of the two brands were evaluated against Pharmacopoeial specification limit.

#### Dissolution profile

Data of the dissolution profile of the two brands were compared using various mathematical models, t-test and model independent approaches (difference factor (f1), similarity factor (f2) and dissolution efficiency (DE)). KinetDS software program was used to determine dissolution efficiency (DE) or the area under a dissolution curve between defined time points and the best fit model for the dissolution profile of both brands. Measures of goodness of fit (Akaike Information Criterion (AIC) and determination coefficient (r^2^)) were used to determine the performance of dissolution models.

## Results

### Assessment of drug efficacy

In total, 679 subjects were recruited of which 418 subjects were enrolled and randomized across the two brands of ABZ (n_Bendex_ = n_Ovis_ = 209). *T*. *trichiura* was the most prevalent (69.4%), followed by *A*. *lumbricoides* (53.6%). Hookworm infections were found in 28.2% of the subjects. In total 388 subjects completed the trial (n_Bendex_ = 197; n_Ovis_ = 191), resulting in a compliance rate of more than 90%. There was no significant difference in mean age (Bendex: 10.3 years *vs*. Ovis: 10.3 years, *p* = 1.00), sex ratio (Bendex: 1.07 *vs*. Ovis: 0.87, *p* = 1.00) and mean fecal egg count (FEC) (*A*. *lumbricoides*: Bendex: 8,706 egg per gram of feces (EPG) *vs*. Ovis: 7,935, *p* = 0.69; *T*. *trichiura*: Bendex: 909 EPG *vs*. Ovis: 769, *p* = 0.45; hookworm: Bendex: 355 EPG *vs*. Ovis: 335, *p* = 0.79) between the two arms at baseline. Both brands showed high efficacy against *A*. *lumbricoides* (Bendex: 98.7% *vs*. Ovis: 97.8%, *p* = 0.64), and poor efficacy against *T*. *trichiura* (Bendex: 24.4% *vs*. Ovis: 20.4%, *p* = 0.81). For hookworm infections, Ovis was more efficacious than Bendex, though the difference was marginally significant (Bendex: 88.7% *vs*. Ovis: 98.1%, *p* = 0.05). Based on the WHO criteria to classify the efficacy of anthelminthic drugs (Table 1), both brands had satisfactory and reduced efficacy against *A*. *lumbricoides* and *T*. *trichiura*, respectively. For hookworms, Ovis had a satisfactory efficacy, whereas Bendex had a doubtful efficacy. A pairwise comparison of baseline parameters and drug efficacy between Bendex and Ovis are presented in S1 Table.

### Assessment of drug quality

#### Mass uniformity

The results of mass uniformity of ABZ tablets of each brand are presented in Table 2. The results revealed that both brands of ABZ tablets complied with pharmacopoeial specification limit [20].

**Table 2:** Results of weight and API-assay of albendazole tablet brands (Bendex and Ovis).

#	Product	Batch no.	Expiry Date	Pack	Weight (mg; mean ± SD) (n = 20)	Assay (%lc; mean ± SD) (n = 6)
**1**	**Bendex**	21253	Nov, 2015	Box of 1x100 tablets	1175.6 ± 4.90	99.10 ± 0.70
**2**	**Ovis**	2020	Nov, 2015	Box of 10x10 tablets	696.7 ± 0.02	99.40 ± 1.30

lc: label claim

#### Amount of active compound

Method validation results indicated the fitness-for-use of the applied HPLC method. The 95% CI for the regression slope equaled 20.47 (95% CI: 20.14 to 20.80) and y-intercept equaled 9.99 (95% CI: -57.44 to 77.41) together with r^2^ value of 0.999 and ANOVA F-value of 17,551 proved a strong positive linear relationship. In addition, random pattern of the residual plot showed a good fit of the linear model to the data. The %RSD (0.26) for repeatability of the method was within the specification limit (%RSD ≤ 0.85). The results of percent recovery (mean % ± %RSD = 99.59 ± 0.57% to 100.42 ± 0.11%) were within acceptable range. The comparative assay results of the two brands of albendazole tablets are presented in Table 2: no significant difference at 95% CI (p ≥ 0.05) was found. Both brands were found to be complying with the acceptance criteria for assay of ABZ tablets [21] *i*.*e*. 90–110% label claim.

#### Dissolution profile

**Fig 2:**
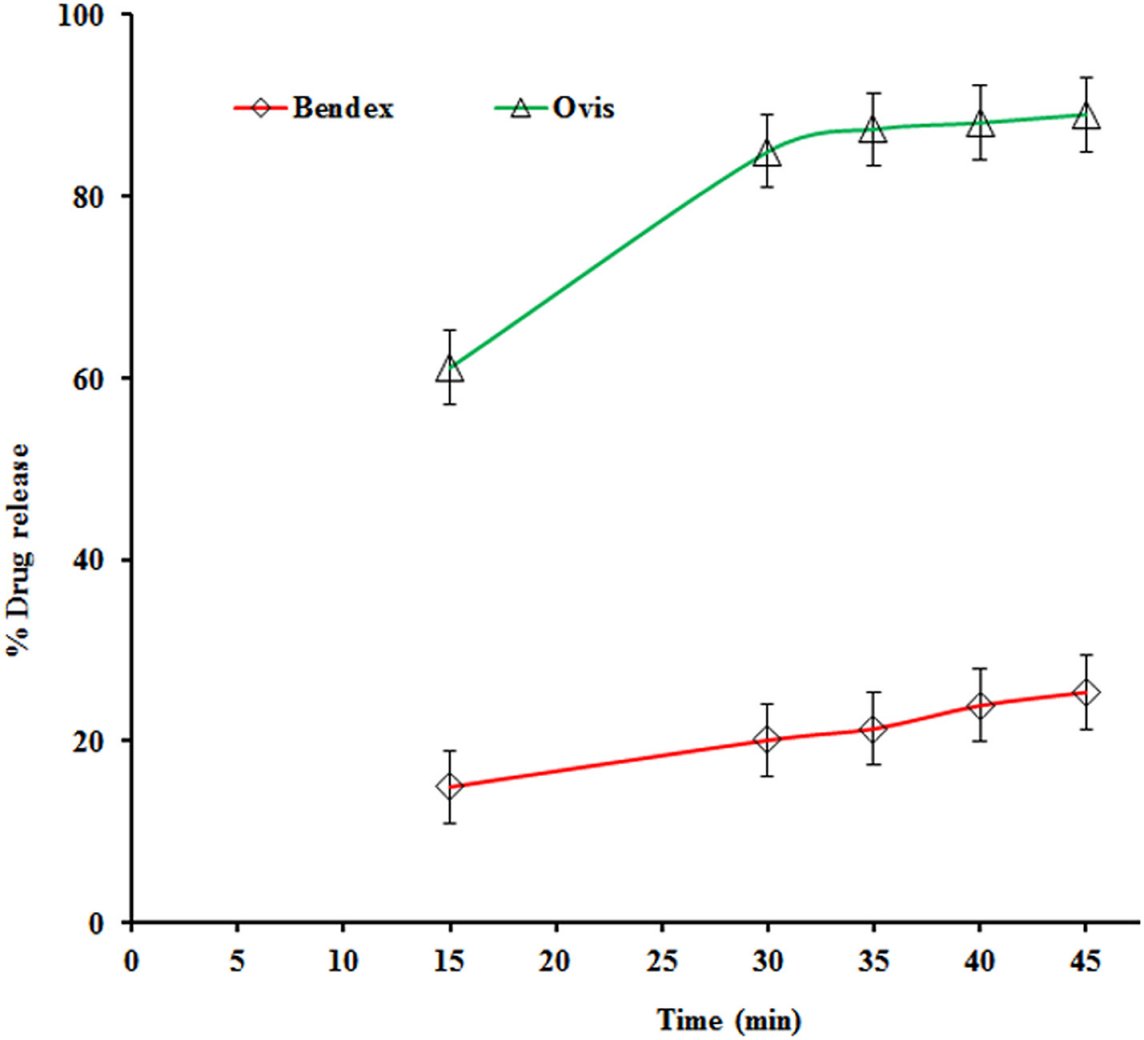
Dissolution profile (mean ± SD, n = 6) of Bendex and Ovis in 0.1 N HCl (900 ml) using USP II apparatus.

The applied method was linear over concentrations ranging from 5 to 15 μg/ml of ABZ. The slope equaled 0.05 (95% CI: 0.04 to 0.05) and intercept equaled 0.02 (95% CI: -0.001 to 0.023) showed linear association between the UV-signal and concentration. In addition, the residual plot indicated a good fit of linear regression. The %RSD for repeatability of the method was 0.01%. The ABZ recovery (mean % ± %RSD) of this method ranged from 98.64 ± 0.00% to 103.18 ± 0.0.01%. The comparative *in-vitro* dissolution results of the two brands of ABZ tablets are presented in Fig 2.

**Fig 3:**
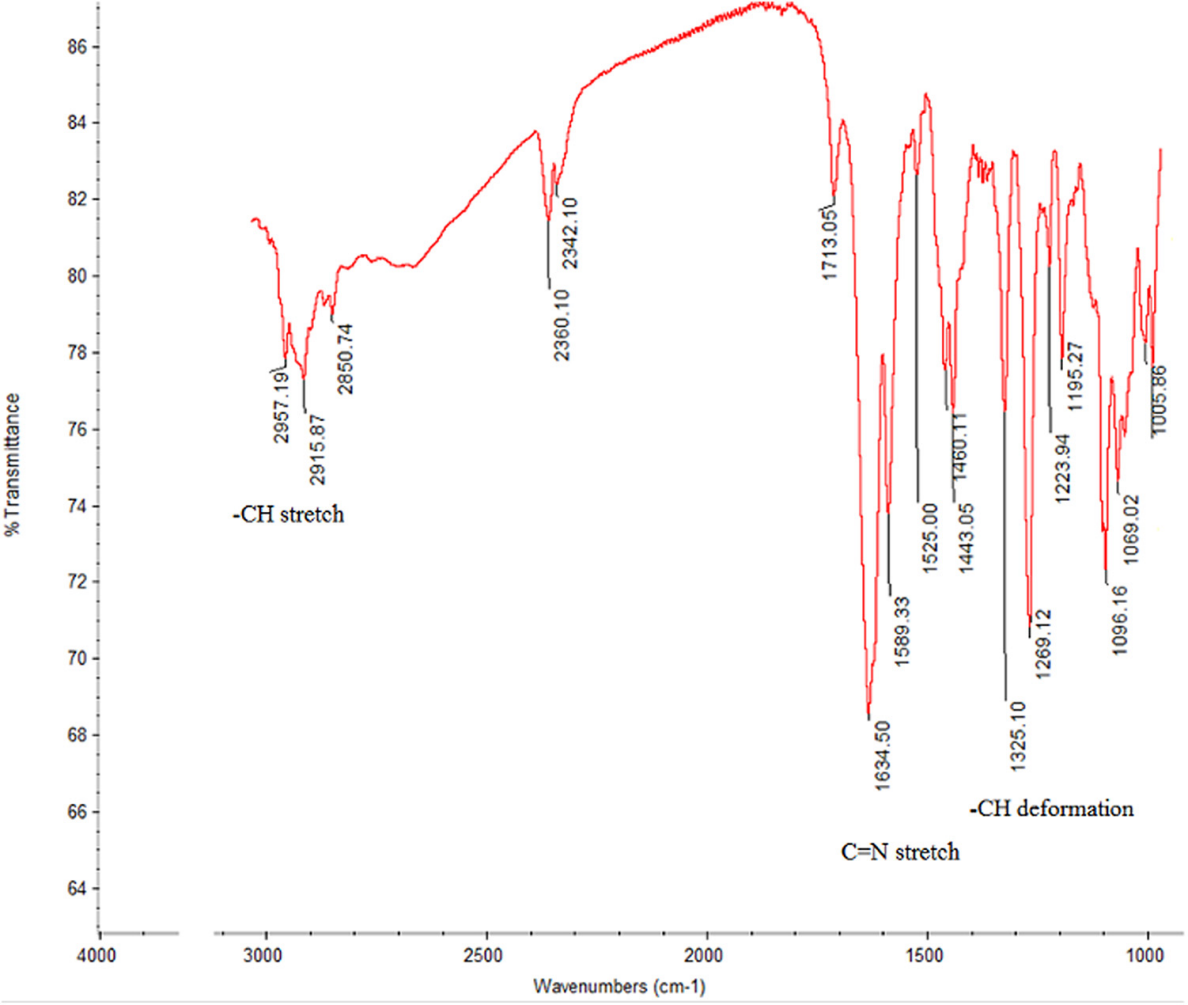
Infra-red spectra of Bendex.

**Fig 4:**
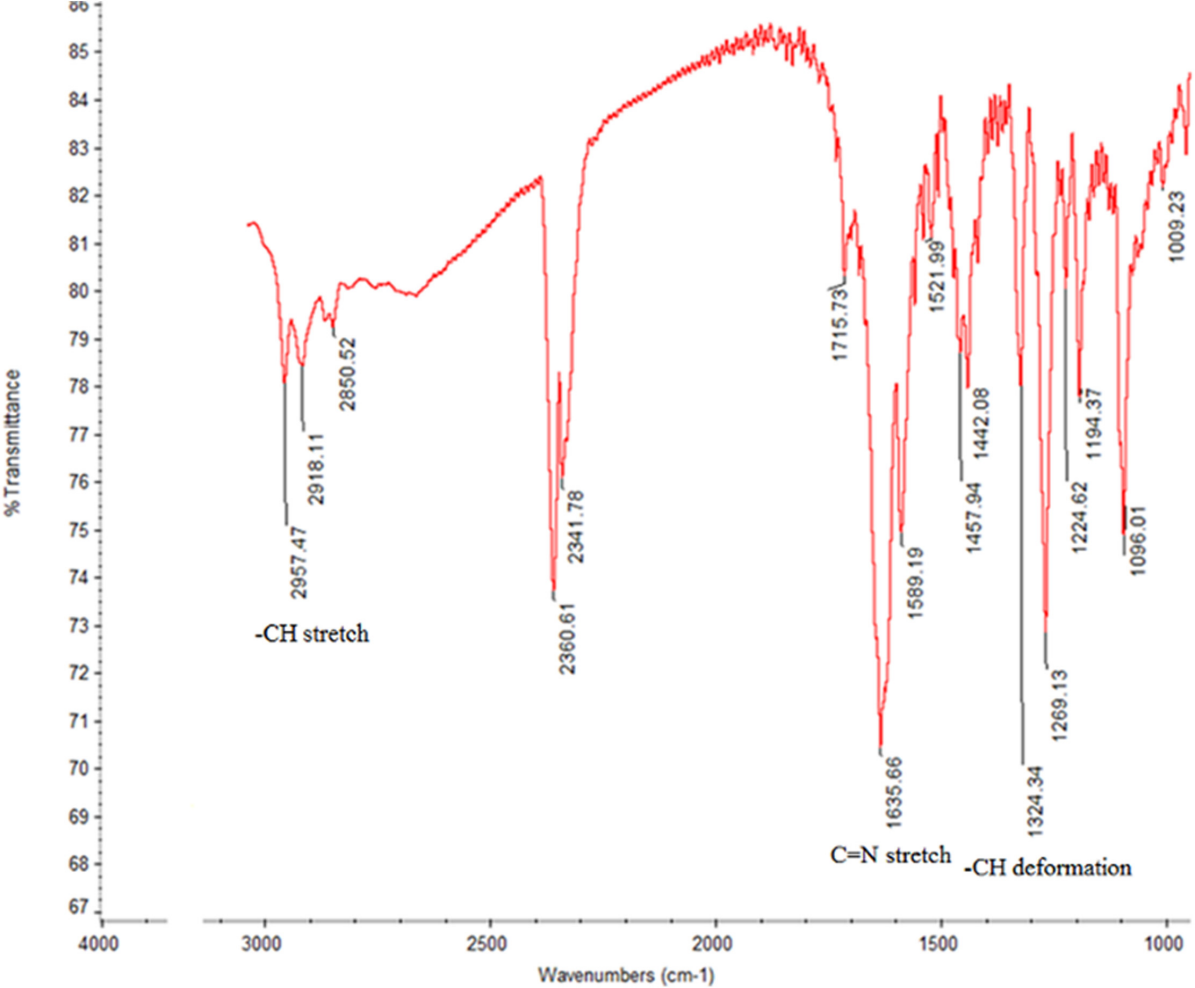
Infra-red spectra of Ovis.

A single-point comparison (amount of dissolved active pharmaceutical ingredient (Q) in 30 minutes) of the two brands of ABZ tablets indicated that Bendex released 20.1%, while Ovis released 84.9%. While Ovis complied with the acceptance criteria of United States Pharmacopoeia (USP) monograph, Bendex failed [21]. The release profile between Bendex and Ovis was significantly (*p* = 0.002) different, as quantitatively expressed by the values of similarity factor (f2) and difference factor (f1) between the two brands being 11 and 74, respectively. The dissolution efficiency (DE) of Bendex (15.83) was lower than the corresponding value observed for Ovis (63.48). The infra-red (IR) spectra of Bendex and Ovis are presented in Figs 3 and 4.

IR spectra of both brands showed no significant difference between the two brands, suggesting the similarity in solid state polymorphic form of ABZ in both brands. The IR spectrum of the major excipient of Ovis (*i*.*e*. anhydrous dibasic calcium phosphate) did not interfere with the ABZ spectrum, while the excipients with interfering IR spectrum (i.e. sodium lauryl sulfate: 2957 and 2918 cm^-1^ and magnesium stearate: 2918 cm^-1^) were only present at much lower concentrations than ABZ in the Ovis tablet, hence neglecting their IR interference. Finally, the release kinetics of ABZ from both brands was evaluated using mathematical modeling. The results of measures of goodness of fit (*i*.*e*. AIC and r^2^) for different dissolution models are presented in S2 Table. The model that best fitted the dissolution data of both brands was Weibull with lag time: the fact that both brands best fitted the data is indicative for a similar release mechanism of albendazole from the tablets.

## Discussion

Assessing the quality and *in-vivo* efficacy differences between different brands of ABZ tablets are very critical to avoid indiscriminate use of different brands that could influence intended therapeutic outcomes. In the present study, we evaluated comparative *in-vivo* efficacy and *in-vitro* quality of two commonly available brands of ABZ tablets (Bendex and Ovis) that are used to treat STH infection.

The *in-vivo* efficacy results of two brands of ABZ against *A*. *lumbricoides* and *T*. *trichiura* determined in terms of ERR, suggest the susceptibility difference between the two STHs. Therapeutic efficacy of antihelmnthics can be influenced by various factors such as infection intensity and susceptibility of parasites. Thus the reduced efficacy of both brands against *T*. *trichiura* observed in the present study could be due to high level of infection intensity [17] and/or genetic modification of beta-tubulin gene [26, 27]. Since concentration of API at the target site of the parasites could be low due to metabolism and/or limited absorption of the drug by the parasite [28, 29], the reduced efficacy of both brands against *T*. *trichiura* might also be associated with the pharmacokinetics of ABZ in the parasite. The reduced efficacy of the two brands against *T*. *trichiura* observed in the present study is comparable to the results reported in the previous studies [6, 17]. The present finding, *i*.*e*. high prevalence of *T*. *trichiura* among other STHs in the study area together with the reduced efficacy results in ERR observed for single dose of ABZ 400 mg tablets, is supported by various literature findings [30–33]. This emphasizes the urgent need for alternative drugs and/or development of novel anthelmintic drugs to tackle this efficacy problem.

Medicines quality is a critical factor that could affect efficacy of drugs against parasites [34] and for biopharmaceutical classification system (BCS) class II [35] drugs like ABZ that have low solubility and high permeability, dissolution is the rate-limiting step for drug absorption. Hence, *in-vivo*/*in-vitro* correlation between blood concentration profile and dissolution profile may be expected. Since the bioavailability of ABZ to the host is very low and also shows variability between individuals [36], a decreased dissolution could significantly worsen bioavailability, which in turn diminishes *in-vivo* efficacy of both the parent drug and therapeutically active metabolite (ABZ sulphoxide). Moreover, the capacity of anthelminthics to dissolve appropriately is an essential characteristic that allows proper drug uptake by the parasites and therefore assures the appropriate drug efficacy. Therefore, the four times decreased dissolution of Bendex compared to Ovis (Fig 2) which could influence both local and systemic concentration is a plausible explanation for the efficacy difference between the two brands against hookworms, which are blood sucking parasites. Previous studies already indicated the differences in uptake and metabolism of benzimidazole drugs among different helminth species [28, 29], which may explain that the efficacy difference between the two brands was only observed against hookworms.

Though mass uniformity and content of API per tablet are critical quality attributes that could influence efficacy, comparable quality of both brands with respect to mass uniformity and ABZ content observed in the present study (Table 2) explain the efficacy difference between the two brands against hookworms is not associated with mass uniformity and content of API.

Considering a single point dissolution specification for ABZ tablets as described in the USP, *i*.*e*. Q ≥ 80% dissolved in 30 min [22], there is a statistically significant (*p* ≤ 0.05) difference observed between Bendex (Q = 20%) and Ovis (Q = 85%) (Fig 2). Also, the area under the dissolution curve between defined time points (0, 15, 30, 45 min) or DE quantifies the poor *in-vitro* dissolution of Bendex. While Ovis thus complied to the USP dissolution specifications, Bendex on the contrary did not. Although an undesirable polymorphic solid state of albendazole could be one of the reasons for the difference in dissolution behavior of API [37–39], the IR spectra (Figs 3 and 4) of Bendex and Ovis showed no significant difference between the two brands. The absence of significant observed shifts in IR-absorption indicates the similarity in polymorphic form of ABZ in both brands. Thus, the significant difference in dissolution observed between the two brands could not be associated with different polymorphic forms of ABZ. Whatever the reason of dissolution difference is, *e*.*g*. excipient and processing manufacturing conditions and stability, the poor dissolution behavior of Bendex observed in the present study is in accordance with the previous reports in which 41 samples out of 72 samples of solid oral dosage forms including ABZ tablets, different generic formulations of albendazole tablets and carbamazepine immediate-release products did not comply with the established acceptance criteria [40–42]. In general, it is important to note that quality of medicines could be one of the factors influencing outcomes of clinical trials. For instance, literature indicates the association of poor quality of locally manufactured antimalarial drugs: Sulfadoxine-Pyrimethamine with clinical failure of malaria treatment in Pakistan [43]. Therefore, the results of the present study and a recent report by Newton and his colleagues [44] point to the requirement of guidelines for quality assurance of medicines used in clinical trials. Subjects lost at follow-up per STH species were the limitations of this study.

In conclusion, this study demonstrated that the two investigated brands of ABZ tablets are efficacious against *A*. *lumbricoides* and hookworm while both brands had reduced efficacy against *T*. *trichiura*. However, there was a significant difference between the two brands of ABZ against hookworm. While both brands showed comparable tablet mass uniformity and albendazole content, the *in-vitro* dissolution release profile between the two brands was significantly different, explaining the clinical efficacy difference observed. The results of the present study underscore the importance of assessing the chemical and physicochemical quality of drugs before conducting efficacy assessment in clinical trials to ensure appropriate therapeutic efficacy and to exclude poor drug quality as a factor of reduced drug efficacy other than anthelminthic resistance. Our *in-vivo* efficacy study clearly indicates the importance of appropriate quality medicines.

## Supporting Information

S1 Checklist CONSORT checklist. (DOC)

S1 Data Raw data of *in-vivo* efficacy study. (XLSX)

S1 Table A pairwise comparison of baseline parameters and drug efficacy between Bendex and Ovis. (DOCX)

S2 Table Mathematical models and corresponding values for measures of goodness of fit. (DOCX)
