# Supplementary material for: Assessment of Efficacy and Quality of Two Albendazole Brands Commonly Used against Soil-Transmitted Helminth Infections in School Children in Jimma Town, Ethiopia
Source: PLoS Negl Trop Dis. 2015 Sep 25;9(9):e0004057. doi: 10.1371/journal.pntd.0004057 (PMC4583991; doi:10.1371/journal.pntd.0004057)
Supplement: S1 Table — (DOCX) [file pntd.0004057.s003.docx]

**S1 Table**. **A pairwise comparison of baseline parameters and drug efficacy between Bendex and Ovis.**

|  | **Bendex** | **Ovis** | ***p*-value** |
| --- | --- | --- | --- |
| Mean age | 10.3 | 10.3 | 1.00 |
| (95% CI) | (9.90; 10.60) | (10.0; 10.60) |  |
| Sex ratio (M:F) | 1.07 | 0.87 | 1.00 |
| (95% CI) | (0.81; 1.43) | (0.65; 1.15) |  |
| ***A. lumbricoides*** | | | |
| N | 106 | 101 |  |
| Mean FEC at baseline (EPG) | 8,706 | 7,935 | 0.69 |
| (95% CI) | (6,357; 11,375) | (5,583; 10,836) |  |
| Mean FEC at follow-up (EPG) | 112 | 175 | 0.66 |
| (95% CI) | (0; 332) | (22; 396) |  |
| ERR (%) | 98.7 | 97.8 | 0.64 |
| (95% CI) | (95.5; 100) | (94.6; 99.7) |  |
| ***T. trichiura*** | | | |
| N | 137 | 129 |  |
| Mean FEC at baseline (EPG) | 909 | 769 | 0.45 |
| (95% CI) | (672; 1,184) | (543; 1,037) |  |
| Mean FEC at follow-up (EPG) | 688 | 612 | 0.66 |
| (95% CI) | (461; 967) | (438; 828) |  |
| ERR (%) | 24.4 | 20.4 | 0.81 |
| (95% CI) | (4.50; 42.20) | (-13.3; 40.9) |  |
| **Hookworm** | | | |
| N | 56 | 56 |  |
| Mean FEC at baseline (EPG) | 355 | 335 | 0.79 |
| (95% CI) | (271; 452) | (234; 449) |  |
| Mean FEC at follow-up (EPG) | 40 | 6 | 0.05 |
| (95% CI) | (9; 88) | (2; 13) |  |
| ERR (%) | 88.7 | 98.1 | 0.05 |
| (95% CI) | (78.7; 97.2) | (96.2; 99.5) |  |
