# Supplementary material for: Assessment of Efficacy and Quality of Two Albendazole Brands Commonly Used against Soil-Transmitted Helminth Infections in School Children in Jimma Town, Ethiopia
Source: PLoS Negl Trop Dis. 2015 Sep 25;9(9):e0004057. doi: 10.1371/journal.pntd.0004057 (PMC4583991; doi:10.1371/journal.pntd.0004057)
Supplement: S2 Table — (DOCX) [file pntd.0004057.s004.docx]

**S2 Table**. **Mathematical models and corresponding values for measures of goodness of fit.**

| **Models** | **Bendex** | | **Ovis** | |
| --- | --- | --- | --- | --- |
|  | **r^2^** | **AIC** | **r^2^** | **AIC** |
| **Zero order** | 0.929 | 24.47 | 0.855 | 46.67 |
| **First order** | 0.673 | 59.55 | 0.642 | 11.29 |
| **Korsmeyer-peppas model** | 0.997 | 27.59 | 0.999 | 49.09 |
| **Weibull** | 0.998 | 25.17 | 0.999 | 29.36 |
| **Weibull with lag time** | 0.999 | 10.76 | 0.999 | 23.49 |
| **Hixson Crowell** | 0.765 | 37.70 | 0.724 | 55.97 |
| **Higuchi** | 0.580 | 43.12 | 0.355 | 65.38 |
| **Korsmeyer-Peppas model with lag time** | 0.999 | 13.99 | 0.999 | 47.00 |
| **Michealis Menten** | 0.999 | 13.76 | 1.000 | 97.18 |
| **Hill equation** | 0.998 | 22.78 | 1.000 | 98.85 |

r^2^: Coefficient of determination, AIC: Akaike Information Criterion (relatively smaller value indicates best fit).
